# Comprehensive Profiling of ac4C RNA Modification Identifies CERCAM in Cancer‐Associated Fibroblasts as a Key Prognostic and Microenvironmental Regulator in Colorectal Cancer

**DOI:** 10.1002/cnr2.70677

**Published:** 2026-09-22

**Authors:** Yang Xia, Jialin Dai, Xinyi Zhou, Ruige Zhang

**Affiliations:** ^1^ Department of General Surgery The Affiliated Hospital With Jiangnan University Wuxi China; ^2^ Department of Pharmacy Kunshan Hospital of Tranditional Chinese Medicine Suzhou China; ^3^ Ansteel General Hospital Anshan China

**Keywords:** ac4C modification, cancer‐associated fibroblasts (CAFs), CERCAM, colorectal cancer, prognostic signature, single‐cell RNA sequencing

## Abstract

**Background:**

N4‐acetylcytidine (ac4C) RNA modification has emerged as a critical epigenetic regulator in tumorigenesis and progression. However, the comprehensive landscape of ac4C modification in colorectal cancer (CRC), particularly its orchestration of the tumor microenvironment (TME) and stromal crosstalk, remains largely unexplored.

**Aims:**

This study aimed to comprehensively delineate the landscape of N4‐acetylcytidine (ac4C) RNA modification in colorectal cancer (CRC) to uncover its role in shaping the tumor microenvironment and clinical outcomes. Integrated bioinformatic and in vitro analyses revealed that ac4C patterns dictate aggressive stromal crosstalk and identified CERCAM as a cancer‐associated fibroblast (CAF)‐specific driver of tumor progression, providing a novel framework for prognostic prediction and personalized targeted therapy.

**Methods:**

Transcriptomic and clinical data of CRC patients were curated from TCGA and GEO databases (GSE14333, GSE17536, GSE38832, GSE39582). The ac4C modification patterns were quantified using single‐sample Gene Set Enrichment Analysis (ssGSEA). Single‐cell RNA sequencing (scRNA‐seq) analyses were performed via the scCancer Explorer database. A prognostic ac4C score was constructed to evaluate clinical outcomes and therapeutic vulnerabilities. The functional role of the identified target gene, CERCAM, was validated using in vitro co‐culture experiments.

**Results:**

Patients with high ac4C scores exhibited worse prognosis, higher clinical stages, and metastasis (N2, M1). High ac4C levels positively correlated with epithelial‐mesenchymal transition (EMT), TGF‐β signaling, and extracellular matrix (ECM) receptor interactions, whereas low ac4C levels were associated with cell cycle, fatty acid metabolism, and microsatellite stability (MSS). The TME of high‐ac4C tumors was characterized by higher Stromal, Immune, and ESTIMATE scores, elevated immune checkpoints (LAG3, CD14, LILRB2, SIRPA, CD8A), and strong correlations with macrophages and NK cells. scRNA‐seq analysis revealed that CERCAM, a key gene within the ac4C network, was predominantly expressed in cancer‐associated fibroblasts (CAFs) rather than tumor cells. DNA methylation of CERCAM was significantly elevated in metastatic CRC compared to primary tumors. In vitro experiments confirmed that CERCAM was overexpressed in CAFs and CRC tissues. Co‐culturing CRC cells (RKO, HCT116) with CERCAM‐knockdown CAFs significantly suppressed tumor cell proliferation, migration, and invasion. Furthermore, the ac4C score effectively predicted tumor mutational landscape differences and sensitivities to agents like pazopanib, gefitinib, and conventional chemotherapies.

**Conclusions:**

This study delineates the crucial role of ac4C modification in shaping the CRC microenvironment, identifies CERCAM as a CAF‐specific driver of tumor progression, and provides a translational framework for ac4C‐based prognostic prediction and personalized targeted therapy.

## 1 Introduction

Colorectal cancer (CRC) remains a formidable global health challenge, ranking as the third most commonly diagnosed cancer and the second leading cause of cancer‐related mortality worldwide [1]. Despite advances in early screening, surgical techniques, and systemic therapies, a substantial proportion of patients with advanced or metastatic disease experience treatment failure. Intratumoral heterogeneity and the dynamic alterations within the tumor microenvironment (TME) are the primary culprits behind therapy resistance and metastatic dissemination [2, 3]. Consequently, deciphering the nuanced molecular layers that drive CRC heterogeneity and stromal interplay is imperative for developing precise prognostic tools and identifying actionable targets.

Recent studies have increasingly highlighted that diverse biological pathways, molecular networks, and epitranscriptomic modifications collectively drive CRC progression and therapeutic resistance [4, 5, 6]. Furthermore, the dynamic crosstalk between these epigenetic alterations and the tumor microenvironment is now recognized as a fundamental pillar of tumor evolution [7]. Epitranscriptomics, particularly RNA modification, has recently reshaped our understanding of post‐transcriptional gene regulation in cancer. Among over 170 known RNA modifications, N4‐acetylcytidine (ac4C), catalyzed mainly by the acetyltransferase NAT10, is the only known acetylation modification on eukaryotic mRNA [8]. Emerging data suggest that ac4C enhances mRNA stability and translational efficiency, thereby governing cell cycle dynamics, metabolic reprogramming, and oncogenesis. Although the dysregulation of specific ac4C regulators, such as NAT10, has been documented in various malignancies, a systematic profiling of the global ac4C modification landscape in CRC—and its specific role in dictating TME architecture and tumor‐stroma crosstalk—remains conspicuously absent. The TME in CRC is a highly dynamic ecosystem, and cancer‐associated fibroblasts (CAFs) are its primary architects, continuously remodeling the ECM and secreting cytokines that cultivate a metastasis‐permissive niche. Bridging the gap between epitranscriptomic alterations, such as ac4C, and CAF‐mediated tumor‐stroma crosstalk represents a vital and unexplored frontier. Increasing evidence indicates that ac4C promotes mRNA stability and translation efficiency, playing pivotal roles in cell cycle regulation, metabolism reprogramming, and tumor progression [9, 10]. While the dysregulation of individual ac4C regulators like NAT10 has been implicated in specific cancers [11], a systematic characterization of the overall ac4C modification landscape—and how it orchestrates the TME and stromal cell interactions in CRC—remains conspicuously missing.

The CRC TME is a complex ecosystem comprising immune cells, extracellular matrix (ECM), and stromal cells, predominantly cancer‐associated fibroblasts (CAFs). CAFs are key architects of ECM remodeling and secrete a plethora of cytokines that foster an immunosuppressive and pro‐metastatic niche [12]. Recent single‐cell RNA sequencing (scRNA‐seq) advancements have provided an unprecedented resolution to decode TME heterogeneity, allowing the identification of cell‐type‐specific drivers that conventional bulk RNA sequencing might obscure [13]. Uncovering how transcriptomic/epigenetic alterations like ac4C are mechanically linked to CAF activation and tumor‐stroma crosstalk represents a critical frontier in CRC research.

In this study, we comprehensively quantified ac4C modification patterns in CRC using single‐sample Gene Set Enrichment Analysis (ssGSEA) based on diverse independent cohorts. By integrating bulk transcriptomics with scRNA‐seq analysis, we identified Cerebral Endothelial Cell Adhesion Molecule (CERCAM)—a gene deeply embedded within the ac4C‐driven network—as a uniquely CAF‐expressed target. Furthermore, through rigorous clinical sample validation and in vitro co‐culture models, we defined the functional role of CAF‐derived CERCAM in driving CRC progression. Our findings provide a novel epitranscriptomic and microenvironmental taxonomy for CRC, offering fresh insights into CAF‐tumor interactions and predicting therapeutic vulnerabilities.

## 2 Methods

### 2.1 Datasets

Transcriptomic profiles with matched clinical annotations for CRC were obtained from the Gene Expression Omnibus (GEO; GSE14333, GSE17536, GSE38832, GSE39582) and The Cancer Genome Atlas (TCGA‐COAD, TCGA‐READ). The TCGA cohort and combined GEO datasets were subjected to batch effect removal using the “ComBat” algorithm via the ‘sva’ R package (v3.46.0). Somatic mutation data were obtained from the TCGA portal. ac4C modification‐related genes were curated from recent literature and established epitranscriptomic databases [8]. Based on the median ssGSEA score serving as the optimal cutoff, patients were stratified into High‐ac4C and Low‐ac4C groups.

### 2.2 ac4C Modification Quantification via ssGSEA

Instead of consensus clustering, we utilized single‐sample Gene Set Enrichment Analysis (ssGSEA) via the “GSVA” R package to quantify the absolute enrichment score of the ac4C modification signature for each patient. Based on the optimal cutoff of the ssGSEA ac4C score, patients were stratified into High‐ac4C and Low‐ac4C groups.

### 2.3 Functional and Pathway Enrichment Analysis

Gene Set Variation Analysis (GSVA) was performed using Hallmark and KEGG gene sets to estimate variations in biological pathways between high and low ac4C groups. The TME landscape was profiled using CIBERSORT for immune cell deconvolution and the ESTIMATE algorithm to compute Stromal, Immune, and ESTIMATE scores. Additional TME subpopulations were quantified using specific marker‐based ssGSEA scoring.

### 2.4 Single‐Cell RNA‐Seq (scRNA‐Seq) Analysis

To investigate the cellular origin of key ac4C‐related targets, we utilized the scCancer Explorer database (https://bianlab.cn/scCancerExplorer/home) [14]. The cell subtype annotations and fibroblast subpopulation classifications were derived from established literature integrated within the database [15]. Uniform Manifold Approximation and Projection (UMAP) was used to visualize cell cluster distributions. Gene expression across distinct cell types was visualized using violin plots and feature plots. DNA methylation levels of the target gene across normal, primary tumor, and metastatic cells were also extracted from matched multi‐omics modules within the database. To ensure data consistency and minimize technical batch effects during CAF subpopulation analysis, only CRC single‐cell datasets sequenced via the 10X Genomics platform were extracted for high‐resolution subclustering. To ensure high fidelity and eliminate batch effects during CAF subpopulation analysis, only scRNA‐seq datasets generated via the 10X Genomics platform were utilized for high‐resolution subclustering.

### 2.5 Mutation and Drug Sensitivity Prediction

Tumor mutation burden (TMB) and the landscape of frequently mutated genes (e.g., APC, TTN) were mapped using the “maftools” package. Drug sensitivity (IC50) for chemotherapeutic and targeted agents was predicted using the “pRRophetic” R package based on the Genomics of Drug Sensitivity in Cancer (GDSC) database. Immune checkpoints (LAG3, CD14, LILRB2, SIRPA, CD8A) were specifically selected based on their established immunosuppressive roles in CRC literature. Stromal, Immune, and ESTIMATE scores were calculated strictly using the ESTIMATE algorithm (v1.0.13). Immune cell infiltration was assessed using the LM22 signature matrix via CIBERSORT (https://cibersortx.stanford.edu/).

### 2.6 Clinical Specimens and Inclusion/Exclusion Criteria

A total of 70 paired fresh primary tumor and matched adjacent normal tissues were collected. The inclusion criteria were: (1) pathologically confirmed primary colorectal adenocarcinoma; (2) no prior neoadjuvant chemotherapy, radiotherapy, or immunotherapy; (3) complete clinicopathological data. Exclusion criteria included: (1) presence of other malignancies; (2) hereditary syndromes (e.g., FAP, Lynch syndrome); (3) history of inflammatory bowel disease. Matched adjacent normal tissues were defined as surgical resection margins located at least 5 cm from the primary tumor edge. To confirm the absence of tumor cell contamination, a fragment of the marginal tissue was histologically examined via H&E staining by two independent pathologists prior to molecular analysis. The study was approved by the local Ethics Committee, and all participants provided written informed consent. Total RNA from 60 tumor tissues showing high relative expression and 10 with low relative expression were selected for subsequent clinical correlations.

### 2.7 qRT‐PCR

Total RNA was extracted using TRIzol Reagent. cDNA was synthesized using the PrimeScript RT Reagent Kit. Quantitative real‐time PCR was performed on a LightCycler 480 System using SYBR Green Premix. Primer sequences were: CERCAM Forward: 5′‐TGGTCCACTCCACCTTCCTT‐3′, Reverse: 5′‐GGCACATTCATGTACCCATAACG‐3′; GAPDH, 5′‐ACAACTTTGGTATCGTGGAAGG‐3′, 3′‐GCCATCACGCCACAGTTTC‐5′. Target gene expression was first normalized to the internal reference (GAPDH) to obtain ΔCt values, and the matched adjacent normal tissue served as the calibrator for the 2^−ΔΔCt^ calculation. The PCR primer sequences used in the research were as follows: CERCAM, 5′‐ACAGCCCTGATGTGGATACC‐3′, 3′‐GCTTCACACTCGGAGTCTGT‐5′.

### 2.8 Cell Culture and co‐Culture System

Human CRC cell lines (RKO, HCT116, SW480) and normal colonic epithelial cells (NCM460) were obtained from the Cell Bank of the Chinese Academy of Sciences (Shanghai, China). Human CRC cell lines (RKO, HCT116, SW480) and normal colonic epithelial cells (NCM460) were obtained from the Cell Bank of the Chinese Academy of Sciences (Shanghai, China). To ensure phenotypic stability and experimental reproducibility, human colorectal CAFs (Sunncell, SNL‐689) and NFs (Procell, CP‐H251) were obtained commercially. These cells were cultured in DMEM supplemented with 10% FBS at 37°C with 5% CO_2_. All cell lines (RKO, HCT116, SW480, NCM460) were STR‐authenticated and routinely tested negative for mycoplasma contamination. For co‐culture experiments, CRC cells (RKO, HCT116) were plated in the lower chambers of Transwell apparatuses, while transfected CAFs were seeded in the upper inserts (0.4 μm pore size), allowing for paracrine signaling and cytokine exchange without direct cellular contact. Short hairpin RNA (shRNA) and overexpression plasmids targeting CERCAM were transfected into CAFs using Lipofectamine 3000. For co‐culture assays, CRC cells (RKO, HCT116) were seeded into the lower chambers of Transwell plates, and transfected CAFs were seeded into the upper inserts (0.4 μm pore size) to allow cytokine/stromal crosstalk without direct physical contact. Specific shRNA sequences targeting CERCAM were GTTTGCAGACACAGACAACAT.

### 2.9 Cell Transfection

Full‐length CERCAM was cloned into the pcDNA3.1(+) vector for overexpression, and shRNA targeting CERCAM was purchased from GenePharma. Cells were transfected using Lipofectamine 3000 (for plasmids) or RNAiMAX (for shRNA). Knockdown and overexpression efficiencies were confirmed 48 h post‐transfection via qRT‐PCR.

### 2.10 Functional Assays (CCK‐8, Scratch, and Transwell Assays)

Post co‐culture, tumor cell proliferation was assessed using the Cell Counting Kit‐8 (CCK‐8) assay at 0, 24, 48, and 72 h. Tumor cell migration was evaluated via the wound‐healing (scratch) assay, measuring closure at 0 and 24 h. Invasion capacity was measured using Matrigel‐coated Transwell inserts (8.0 μm pore size), where tumor cells co‐cultured with CAFs were allowed to invade for 24 h before staining and quantification.

### 2.11 Data Processing and Differential Expression Analysis

Transcriptomic data from TCGA and GEO datasets were strictly normalized to log_2_(TPM + 1) prior to analysis. The limma R package with voom precision weights was used to identify differentially expressed genes (DEGs) between the high and low ac4C groups. A moderated *t*‐test with Benjamini–Hochberg (BH) correction was applied, and DEGs were defined by |log_2_ fold change (FC)| > 0.5 and adjusted *p* < 0.05.

### 2.12 Statistical Analysis

Analyses were performed using R (v4.2.1) and GraphPad Prism. Continuous variables were compared using Student's *t*‐test or Wilcoxon rank‐sum tests. Survival curves were generated using the Kaplan–Meier method with log‐rank tests. Differences in proportions were assessed via Chi‐square tests. *p* < 0.05 was considered statistically significant. For analyses involving multiple comparisons, *p*‐values were adjusted using the False Discovery Rate (FDR) correction.

## 3 Results

### 3.1 Quantification of ac4C Modification Patterns and Molecular Stratification in CRC

**FIGURE 1:**
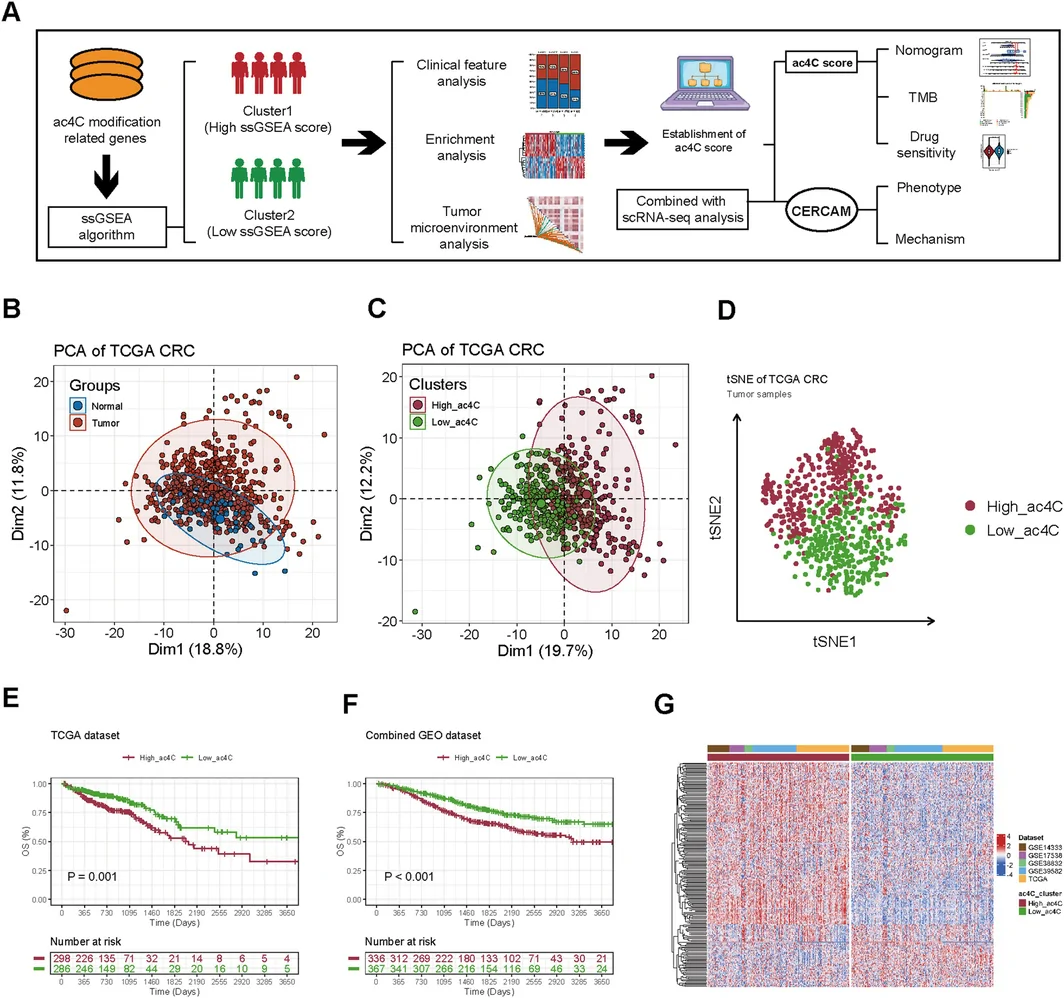
Quantification of ac4C modification patterns and molecular stratification in colorectal cancer (CRC). (A) Schematic flowchart illustrating the overall design and analysis pipeline of this study. (B) Heatmap showing the expression landscape of ac4C modification‐related genes, distinguishing normal colonic tissues from CRC samples in the TCGA cohort. (C and D) Principal component analysis (PCA) and t‐distributed stochastic neighbor embedding (t‐SNE) (D) plots demonstrating distinct transcriptional separation between ac4C Cluster 1 and Cluster 2. (E and F) Kaplan–Meier overall survival curves comparing CRC patients in Cluster 1 and Cluster 2 (log‐rank test, *p* < 0.05). (G) Alluvial diagram illustrating the consistent distribution of the two ac4C‐associated clusters across four independent GEO datasets (GSE14333, GSE17536, GSE38832, and GSE39582).

The overall design of this study is summarized in Figure 1A. We first evaluated the baseline expression landscape of ac4C regulators. While normal colonic tissue samples are naturally underrepresented, PCA revealed a distinct separating trend between normal and CRC tissues, confirming that the overall transcriptomic profile of ac4C regulators is fundamentally altered during tumorigenesis (Figure 1B). The clinical characteristics of patients in each cluster are detailed in Table S1 (All CRC patients' clinical characteristics). ac4C modification‐related genes are summarized in Table S2 (ac4C modification‐related genes). To further explore biological heterogeneity, we calculated a continuous ac4C modification score for each patient using ssGSEA. By stratifying the cohort based on the median score, we robustly identified two distinct molecular subgroups, designated as the high‐ac4C group (Cluster 2) and the low‐ac4C group (Cluster 1).

The stability and transcriptional distinctness of this classification were further validated. Principal component analysis (PCA) and t‐distributed stochastic neighbor embedding (t‐SNE) demonstrated a clear spatial separation between the two clusters, confirming that ac4C modification patterns define coherent molecular subtypes (Figure 1C,D, Figure S1A,B). Survival analysis revealed a significant prognostic divergence between these subgroups, with Cluster 2 exhibiting markedly worse overall survival compared to Cluster 1 (*p* < 0.05; Figure 1E,F). To ensure the robustness of our classification, we extended this analysis to multiple external validation sets. An alluvial diagram or composition plot illustrated the consistent distribution of these two ac4C‐associated clusters across four independent GEO datasets (GSE14333, GSE17536, GSE38832, and GSE39582), confirming the stability of ac4C‐based subtyping across different populations and platforms (Figure 1G).

### 3.2 Clinical and Pathological Correlation of ac4C Modification Levels

**FIGURE 2:**
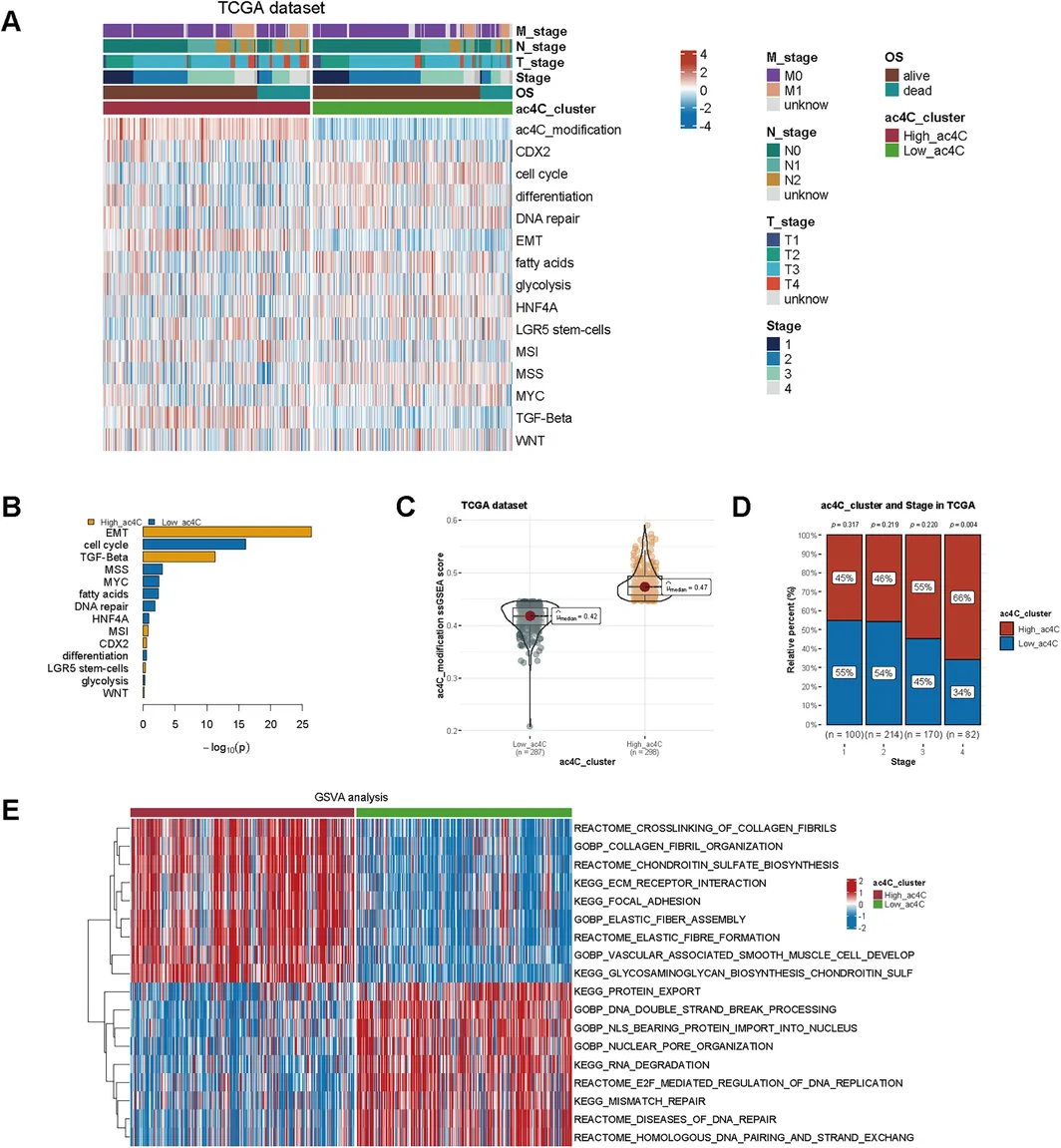
Clinical, pathological, and pathway characteristics of ac4C modification patterns. (A and B) Distribution of specific clinicopathological features showing the enrichment of advanced N‐stage (N2) (A) and distant metastasis (M1) (B) in the high ac4C group/Cluster 2. (C) Comparison of ac4C modification ssGSEA scores between Cluster 1 and Cluster 2 in the TCGA cohort. (D) Proportion of patients with high vs. low ac4C modification levels across advancing overall pathological stages. (E) Pathway enrichment analyses depicting pathways significantly correlated with high ac4C scores (e.g., EMT, TGF‐β signaling) and low ac4C scores.

As shown in Figure 2, clinical correlation analysis revealed that the high ac4C group was significantly enriched for more aggressive clinicopathological features. Specifically, a high ac4C score or Cluster 2 membership was closely associated with advanced N‐stage (N2) and distant metastasis (M1) (Figure 2A,B, Figure S2A,B). The differentially expressed genes identified between the two ac4C modification subtypes are presented in Table S3 (DEGs between Cluster 1 and Cluster 2). To quantify these patterns at an individual level, we utilized ssGSEA and confirmed that Cluster 1 had a significantly lower ac4C modification ssGSEA score than Cluster 2 according to the TCGA database (Figure 2C). However, the difference was not significant in the combined GEO database (Figure S2C). Furthermore, we observed a progressive increase in the proportion of high‐ac4C patients as the overall pathological stage of the tumor advanced, indicating that ac4C modification levels may accumulate during CRC progression (Figure 2D, Figure S2D).

Pathway enrichment analyses indicated profound biological divergence between the groups. High ac4C scores were strongly and positively correlated with malignant phenotypic pathways, including Epithelial‐Mesenchymal Transition (EMT) and TGF‐β signaling. Conversely, low ac4C scores were positively correlated with cell cycle regulation, fatty acid metabolism, and pathways associated with microsatellite stability (MSS) (Figure 2E).

### 3.3 High ac4C Drives a Fibrotic and Immunosuppressive TME

**FIGURE 3:**
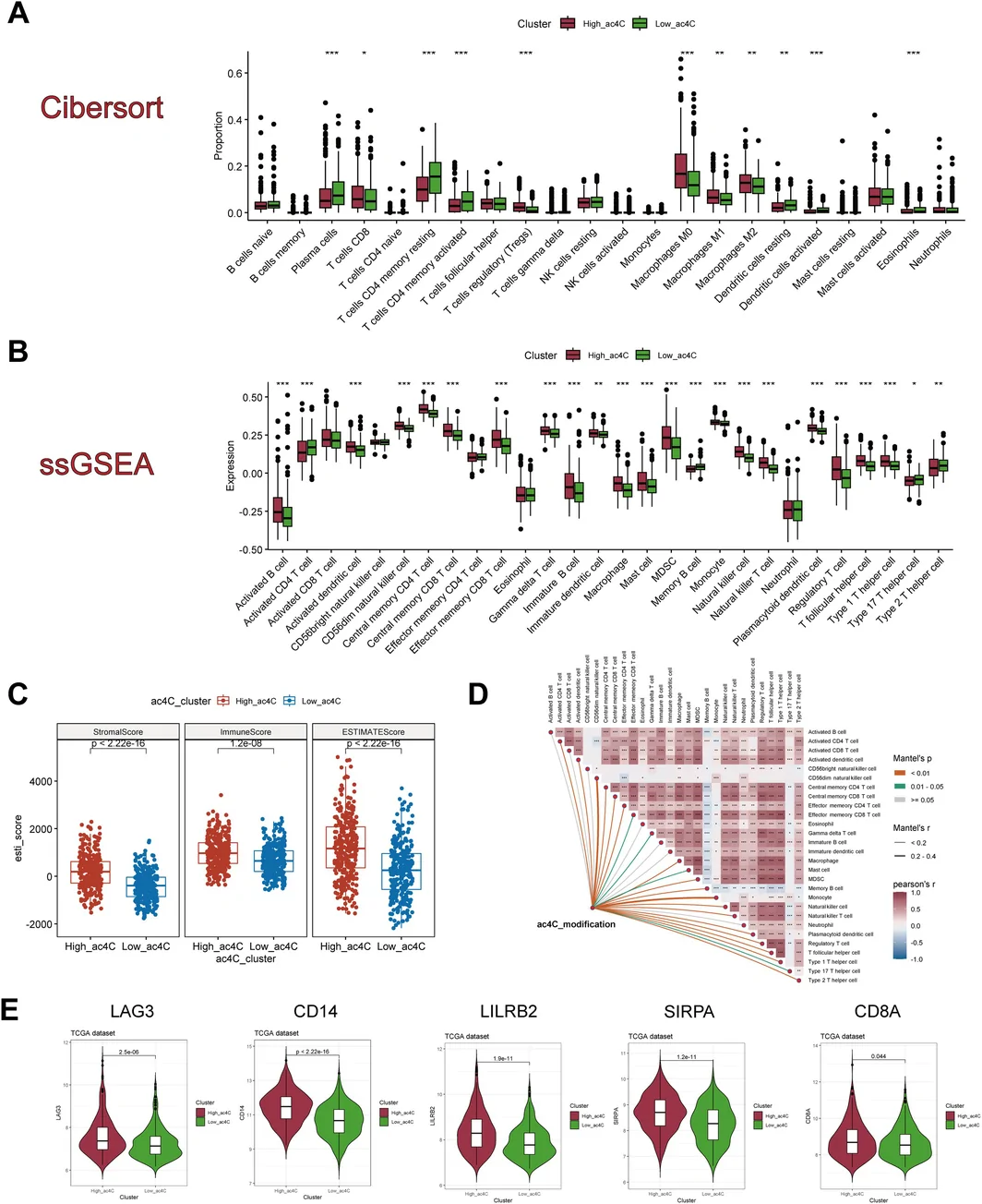
ac4C levels sculpt an immunosuppressive and fibrotic tumor microenvironment. (A) Relative infiltration proportions of immune cells estimated by CIBERSORT, comparing the high and low ac4C groups. (B) Single‐sample gene set enrichment analysis (ssGSEA) scores for various immune cell types (e.g., activated B cells, NKT cells, regulatory T cells) between the two groups. (C) Comparison of Stromal, Immune, and ESTIMATE scores between high and low ac4C tumors using the ESTIMATE algorithm. (D) Correlation network depicting the relationships between the ac4C score and innate immune components (NK cells, monocytes, and macrophages). (E) Expression levels of key immune checkpoint molecules and inhibitory receptors (LAG3, CD14, LILRB2, SIRPA, and CD8A) in the high vs. low ac4C groups.

Given the enrichment in ECM and EMT pathways, we deeply profiled the TME (Figure 3). CIBERSORT analysis revealed that low‐ac4C tumors contained more abundant plasma cells and CD4+ T cells, but significantly fewer macrophages compared to high‐ac4C tumors (Figure 3A, Figure S3A). Secondary independent verification using ssGSEA confirmed that the low ac4C group possessed fewer activated B cells, NKT cells, and regulatory T cells (Figure 3B, Figure S3B). Notably, ESTIMATE algorithm analyses demonstrated that high‐ac4C tumors exhibited significantly higher Stromal, Immune, and ESTIMATE scores, indicative of a dense, stroma‐rich microenvironment (Figure 3C, Figure S3C). Correlation networks demonstrated that the ac4C score was most significantly associated with innate immune components, specifically NK cells, monocytes, and macrophages (Figure 3D, Figure S3D). This was accompanied by a robust upregulation of key immune checkpoint molecules and inhibitory receptors in the high‐ac4C group, specifically LAG3, CD14, LILRB2, SIRPA, and CD8A (Figure 3E, Figure S3E). Together, these data suggest that high ac4C modification drives an immuno‐exhausted and highly fibrotic TME.

Furthermore, high‐ac4C tumors exhibited robust upregulation of critical immune checkpoint and inhibitory molecules, including LAG3, CD14, LILRB2, SIRPA, and CD8A. Collectively, these findings suggest that ac4C overactivity actively fosters a stroma‐rich, immune‐exhausted, and fibrotic niche.

### 3.4 Prognostic Value of the ac4C Score and Nomogram Construction

**FIGURE 4:**
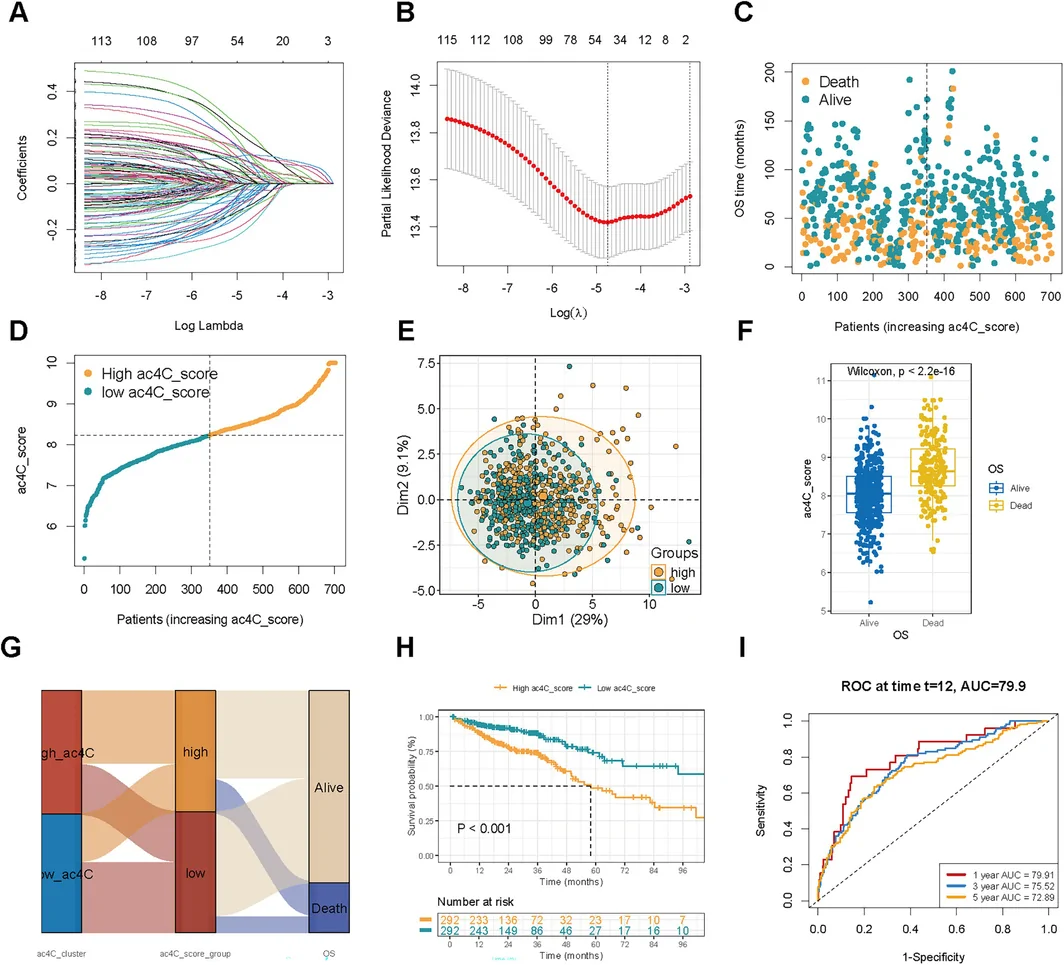
Development and validation of the ac4C Prognostic Signature. (A and B) Construction of the prognostic signature using LASSO‐Cox regression. (A) LASSO coefficient profiles of the ac4C modification‐related genes. (B) Ten‐fold cross‐validation for tuning parameter (λ) selection in the LASSO model. (C) Kaplan–Meier survival analysis for patients stratified into high and low ac4C Score groups in the TCGA training cohort. (D) Distribution of ac4C Scores between surviving and deceased patients in the training cohort (Wilcoxon test). (E) Survival analysis evaluating the prognostic value of the developed ac4C signature. (F) Alluvial diagram illustrating the associations among ac4C modification subtypes (Cluster 1 vs. 2), ac4C Score groups (High vs. Low), and patient survival status (Alive vs. Dead). (G) The relationship among different ac4C clusters, high‐ vs. low‐ac4C score groups, and corresponding prognostic outcomes. (H and I) Predictive performance of the ac4C Score. (H) Kaplan–Meier curves for the high‐ and low‐score groups in an independent validation cohort. (I) Corresponding time‐dependent receiver operating characteristic (ROC) curves of the ac4C score for survival prediction.

To construct a clinically applicable prognostic model, we intersected the differentially expressed genes between the high‐ and low‐ac4C groups with core ac4C regulators. Subsequently, LASSO‐Cox regression analysis was utilized to screen and identify a robust gene signature associated with overall survival (Figure 4A). The distribution of the ac4C scores and the corresponding survival status scatter plots further visualized this trend, clearly demonstrating that mortality events were predominantly concentrated in the high‐score demographic (Figure 4B,C). The gene model used to calculate the ac4C modification score was presented in Table S4 (The gene model constituting ac4C modification score).

Kaplan–Meier survival analyses firmly established the prognostic efficacy of this stratification. Consistent with our initial findings, a high ac4C score served as a powerful predictor of poor overall survival, a result that was highly reproducible across both the training (TCGA) and external validation (combined GEO) cohorts (Figure 4D–F, Figure S4A–D). To elucidate the interrelationship between the previously defined molecular clusters, the continuous ac4C score, and ultimate survival outcomes, an alluvial diagram was constructed. This analysis revealed a striking clinical trajectory: there was a predominant flow of patients from ac4C Cluster 1 to the low ac4C score group and subsequently to favorable survival outcomes. Conversely, patients categorized in ac4C Cluster 2 predominantly transitioned to the high ac4C score group, ultimately correlating with mortality (Figure 4G).

**FIGURE 5:**
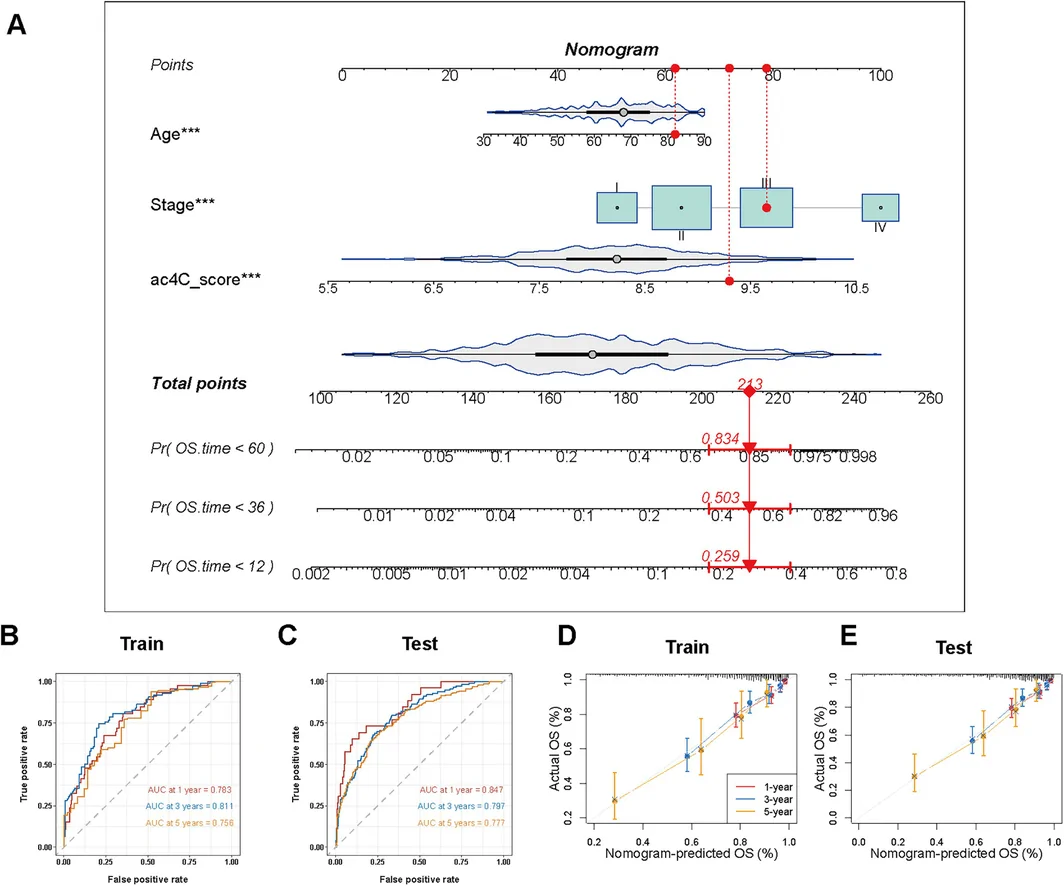
Construction and assessment of a predictive nomogram integrating the ac4C score. (A) A prognostic nomogram incorporating standard clinicopathological features and the ac4C score for predicting 1‐, 3‐, and 5‐year overall survival probabilities. (B–E) Calibration curves comparing the nomogram‐predicted versus observed overall survival rates at 1‐, 3‐, and 5‐year time points.

Finally, the predictive accuracy of the ac4C scoring system was rigorously evaluated using time‐dependent Receiver Operating Characteristic (ROC) analyses. The corresponding ROC curves yielded high Area Under the Curve (AUC) values across multiple time points (e.g., 1‐, 3‐, and 5‐year survival), demonstrating the robust sensitivity and specificity of the ac4C score in predicting long‐term outcomes for CRC patients (Figure 4H,I, Figure S4E,F). To facilitate clinical translation, we integrated the ac4C score with standard clinicopathological features to construct a Nomogram. The Nomogram demonstrated excellent predictive accuracy for 1‐, 3‐, and 5‐year survival, with calibration curves showing high concordance between predicted and observed outcomes, outperforming traditional TNM staging alone (Figure 5A–E).

### 3.5 Genomic Alterations and Drug Sensitivity Associated With ac4C

**FIGURE 6:**
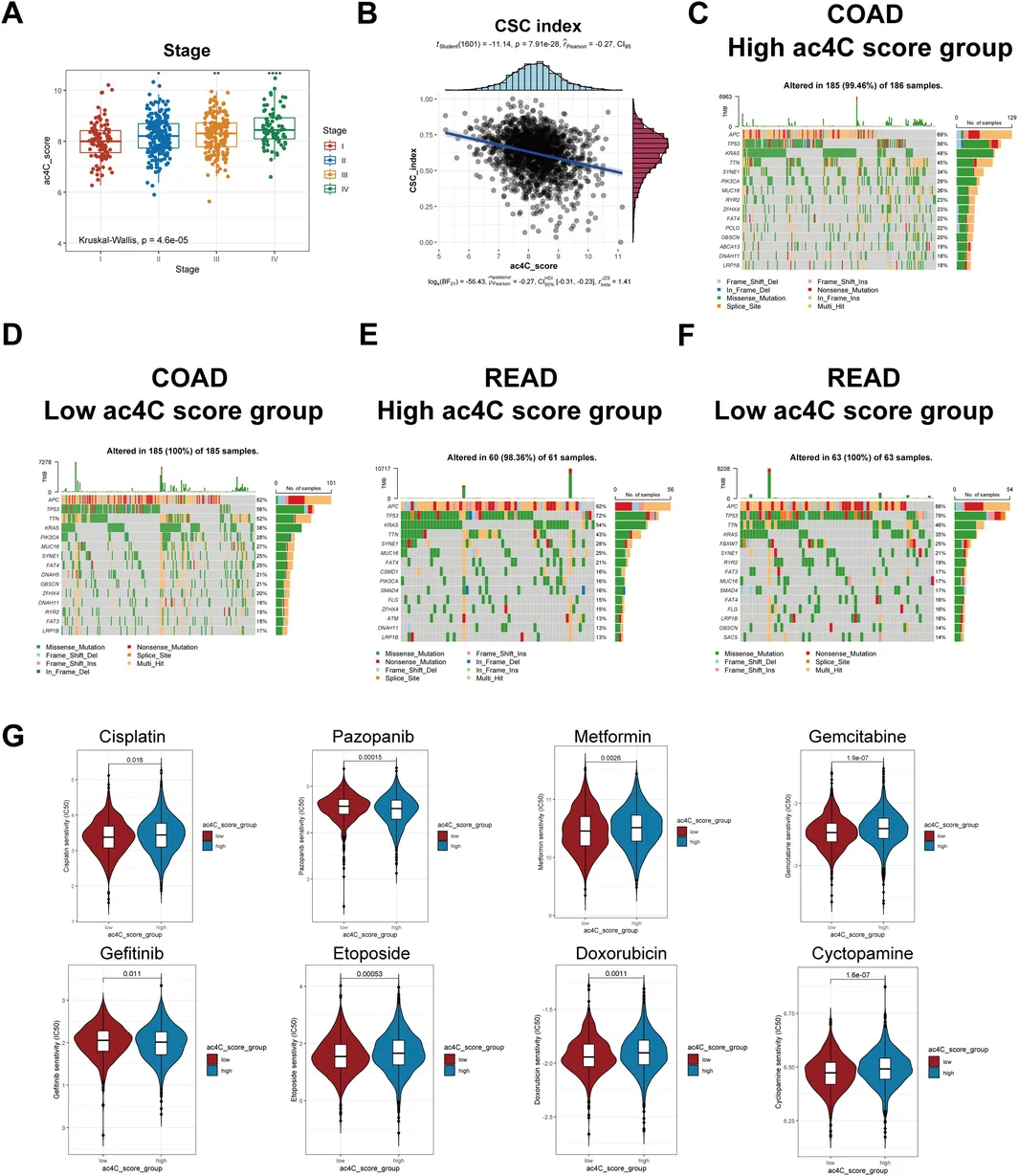
Genomic alterations and therapeutic implications associated with the ac4C score. (A) Comparison of ac4C scores across advancing clinical TNM stages. (B) Correlation between the ac4C score and the Cancer Stem Cell (CSC) index. (C–F) Mutational landscape and spatial heterogeneity analysis. Oncoplots and specific mutation frequencies demonstrate differing patterns of key gene mutations (e.g., APC and TTN) between high and low ac4C score groups in the COAD cohort vs. the READ cohort. (G) Predicted drug sensitivity analysis (IC_50_ values) highlighting differential sensitivities to targeted therapies (pazopanib, gefitinib) and conventional agents (cisplatin, metformin, gemcitabine) between the two groups.

We further investigated whether ac4C scores align with genomic mutations and therapeutic vulnerabilities (Figure 6). Consistent with clinical data, patients at later TNM stages had significantly higher ac4C scores (Figure 6A). Cellular stemness potential, as measured by the CSC index, was significantly and inversely correlated with prognostic signature values (Figure 6B). Based on the median ssGSEA score of ac4C regulators, patients were stratified into high and low ac4C groups. We explored the mutational landscape differences between these groups. Using Fisher's exact test (Table S5), we observed that KRAS mutation frequencies showed a significant *p*‐value difference between subgroups; however, the FDR was not significant, indicating that while mutational disparities may exist, larger cohorts are required for definitive validation. Interestingly, the mutational landscape exhibited spatial heterogeneity: in the COAD strictly colon cohort, patients with high ac4C scores showed significantly lower mutation frequencies of APC and TTN compared to the low ac4C group. In stark contrast, in the READ (rectal cancer) cohort, the high ac4C score group displayed significantly higher APC and TTN mutation frequencies, highlighting distinct epigenetic‐genetic interactions between colon and rectal segments (Figure 6C–F). Interestingly, spatial genomic heterogeneity was observed: within the strict colon cancer cohort (COAD), high ac4C scores correlated with lower mutation frequencies of APC and TTN. In stark contrast, in the rectal cancer cohort (READ), high ac4C scores were associated with significantly higher mutation frequencies of these genes.

Predictive pharmacogenomics revealed profound differences in drug sensitivity. Patients with high ac4C scores were predicted to be significantly more sensitive to targeted therapies such as pazopanib and gefitinib. However, they exhibited marked resistance (lower sensitivity) to conventional chemotherapeutics and other agents, including cisplatin, metformin, gemcitabine, etoposide, doxorubicin, and cyclopamine, compared to the low ac4C group (Figure 6G). Drug sensitivity prediction via oncoPredict suggested that the high ac4C group might be more sensitive to specific targeted inhibitors like pazopanib. Although these agents are not standard first‐line CRC treatments, our findings serve as a hypothesis‐generating exploration for potential targeted therapy candidates.

### 3.6 scRNA‐Seq Identifies CERCAM as an ac4C‐Associated Key Target in CAFs

**FIGURE 7:**
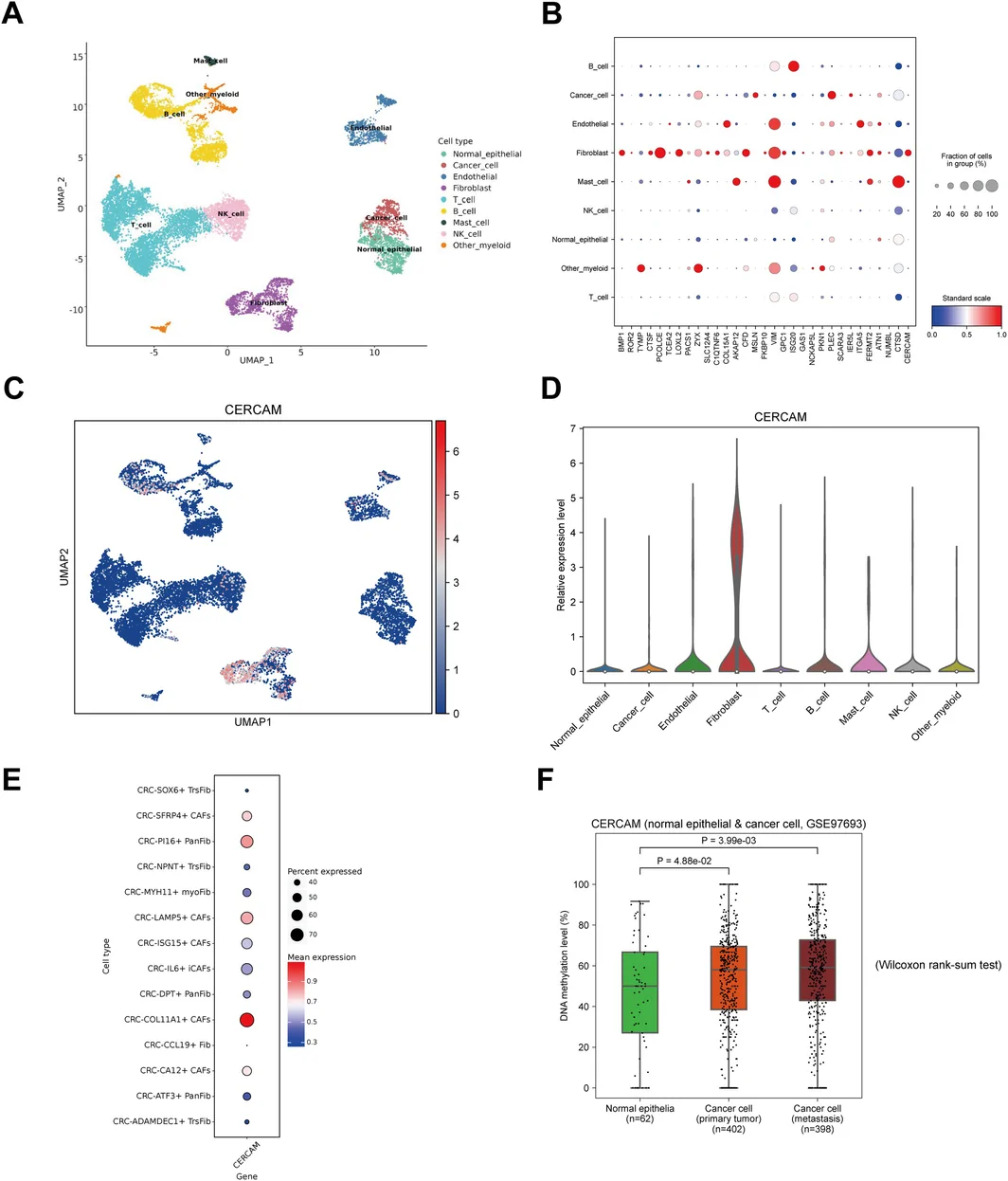
Single‐cell RNA‐seq identifies CERCAM as an ac4C‐associated key target in specific CAF subpopulations. (A) UMAP plot annotating diverse tumor microenvironment (TME) cell populations derived from the scCancer Explorer database. (B) Dot plot showing the expression of canonical marker genes used for cell clustering. (C and D) Feature plot (C) and Violin plot (D) revealing the exclusive and high expression of CERCAM within the general Cancer‐Associated Fibroblasts (CAFs) cluster. (E) Violin plots based on high‐resolution subclustering of 10X Genomics datasets, demonstrating the specific enrichment of CERCAM in matrix‐remodeling CAF subpopulations (myCAFs). (F) Comparison of CERCAM DNA methylation levels across normal cells, primary CRC cells, and metastatic CRC cells.

To understand the cellular origin of the dense stroma and ECM signaling driven by high ac4C, we mined the single‐cell database scCancer Explorer (Figure 7). Cell clustering successfully annotated diverse TME populations (Figure 7A). The markers were shown in Figure 7B. Among the prognostic genes identified by the LASSO‐Cox model, CERCAM was prioritized for further validation because it possessed the highest coefficient weight (*β* = 0.5317), contributing most significantly to the risk formula. Furthermore, epigenetic analysis revealed hypomethylation specifically in the promoter region of CERCAM. Interrogating ac4C‐network hub genes revealed that CERCAM was exclusively and highly expressed in the general Cancer‐Associated Fibroblasts (CAFs) cluster, with virtually no expression in epithelial tumor cells, T cells, or B cells (Figure 7C,D).

To prevent batch effects, we restricted our high‐resolution fibroblast subclustering to 10X Genomics datasets. Violin plots revealed that CERCAM is not a ubiquitous fibroblast marker but is specifically enriched in active, matrix‐remodeling subpopulations, particularly myofibroblastic CAFs (myCAFs), while remaining low in inflammatory CAFs (iCAFs) and normal fibroblasts. Given the profound functional heterogeneity of the tumor stroma, we further deeply dissected the specific fibroblast subpopulations responsible for CERCAM expression. To minimize technical batch effects, we integrated and filtered all available CRC single‐cell datasets generated exclusively via the 10X Genomics platform. High‐resolution subclustering of the fibroblast lineage and subsequent violin plots (Figure 7E) demonstrated that CERCAM is not uniformly expressed across all fibroblasts in the CRC microenvironment. Instead, its expression was robustly and specifically enriched in matrix‐remodeling CAF subpopulations—such as myofibroblastic CAFs (myCAFs)—while remaining relatively low in inflammatory CAFs (iCAFs) and normal resting fibroblasts. This suggests that CERCAM is a distinctive marker for functionally active, matrix‐producing CAF states in colorectal cancer. Epigenetically, we assessed the DNA methylation status of CERCAM. Strikingly, CERCAM methylation levels were highest in metastatic CRC cells, intermediate in CRC cells, and lowest in normal cells (Figure 7F), suggesting a dual epigenetic regulation (RNA modifying and DNA methylation) module underlying its expression during CRC progression.

### 3.7 CAF‐Derived CERCAM Promotes CRC Cell Proliferation and Migration In Vitro

**FIGURE 8:**
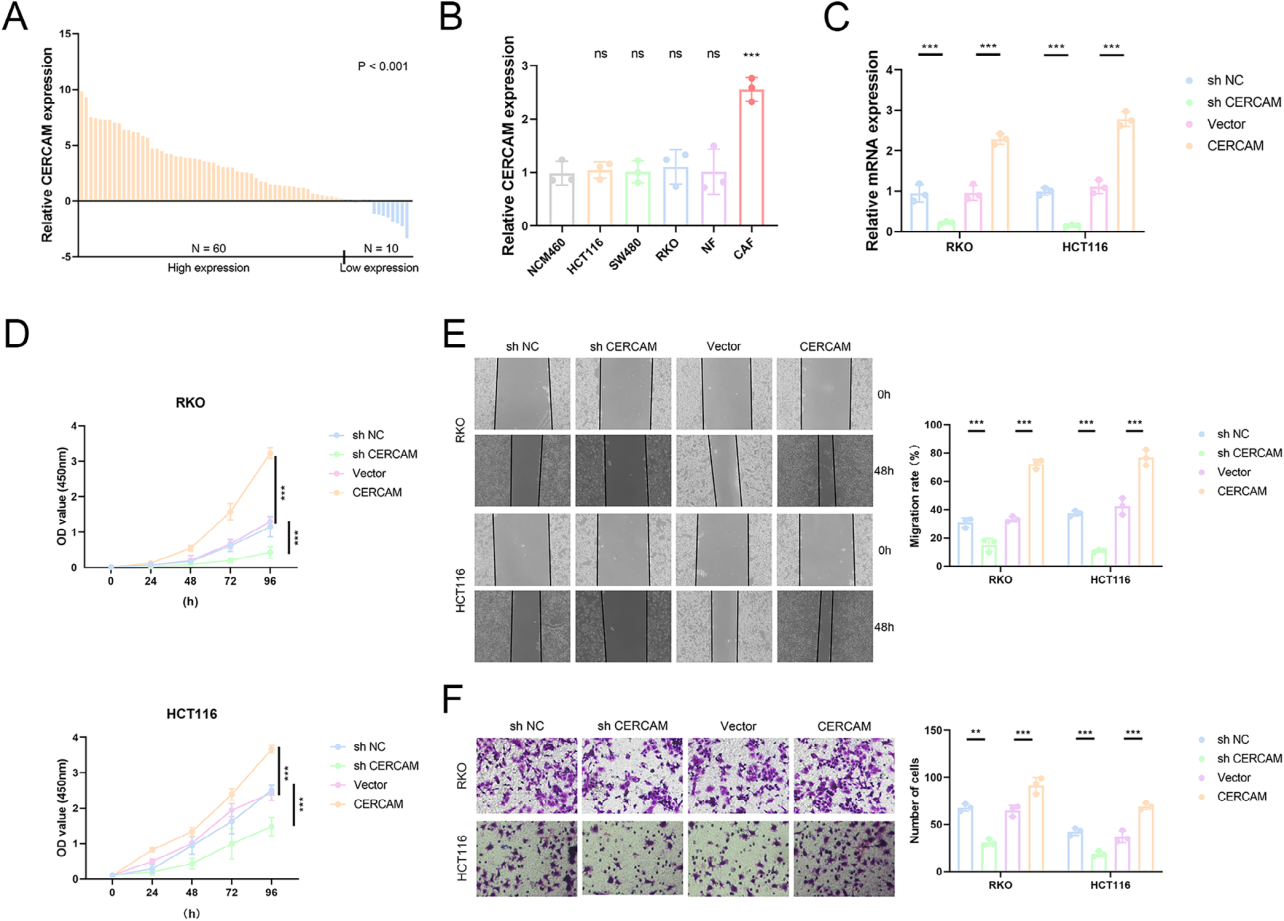
CAF‐derived CERCAM promotes CRC cell proliferation, migration, and invasion in vitro. (A) Relative expression of CERCAM in 70 fresh CRC clinical samples (High CERCAM expression, *n* = 60; Low CERCAM expression, *n* = 10). (B) RT‐qPCR quantification of CERCAM expression across various cell lines (CAFs, NFs, RKO, HCT116, SW480, and NCM460). (C) RT‐qPCR validation of CERCAM knockdown and overexpression efficiency in CAFs. (D) CCK‐8 proliferation assays of RKO and HCT116 tumor cells co‐cultured with control or CERCAM‐modified CAFs. (E and F) Wound‐healing (scratch) assays (E) and Transwell invasion assays (F) assessing the impact of CAF‐derived CERCAM on the migratory and invasive capacities of co‐cultured CRC cells.

Finally, we validated the clinical prevalence and functional necessity of CERCAM (Figure 8). In our clinical cohort of 70 paired samples, 60 patients exhibited higher CERCAM expression in tumor tissues compared to their own matched adjacent normal tissues, whereas 10 showed equal or lower expression, confirming an overall trend of upregulation in CRC (Figure 8A). Subsequent RT‐qPCR across cell lines (CAFs, NFs, RKO, HCT116, SW480, NCM460) definitively validated our scRNA‐seq findings: CERCAM expression in CAFs was dramatically higher than in normal fibroblasts and all other epithelial/tumor cell lines (Figure 8B).

To functionally test whether CAF‐derived CERCAM influences tumor cells, we knocked down or overexpressed CERCAM in CAFs and established a Transwell co‐culture system with RKO and HCT116 tumor cells. The knockdown and overexpression efficiencies of CERCAM were verified by RT‐qPCR (Figure 8C). CCK‐8 assays revealed that CRC cells co‐cultured with CERCAM‐knockdown CAFs exhibited significantly impaired proliferation rates (Figure 8D). Similarly, wound‐healing (scratch) and Transwell invasion assays demonstrated that depleting CERCAM in neighboring CAFs remarkably attenuated the migratory and invasive capacities of the co‐cultured CRC cells (Figure 8E,F).

## 4 Discussion

Our comprehensive investigation systematically unravels the impact of ac4C RNA modification on the progression and microenvironment of CRC. Through ssGSEA quantification, we established an ac4C score that robustly predicts patient prognosis, clinical stage, and responses to systemic therapies. Our analysis definitively mapped CERCAM expression almost exclusively to CAFs. While CERCAM is deeply embedded within the ac4C‐associated transcriptional network, it is important to note that direct acetylation of CERCAM mRNA by NAT10 in CAFs has not yet been experimentally validated. The interplay between ac4C regulators and CERCAM may be mediated via indirect downstream cytokine loops or secondary stroma‐epithelium crosstalk. Future mechanistic investigations utilizing ac4C‐RIP‐seq are required to establish whether NAT10 directly modifies CERCAM transcripts.

The clinical relevance of the ac4C modification in our study is striking. High ac4C levels were firmly linked to aggressive phenotypes, including N2 and M1 stages, as well as canonical metastatic pathways like EMT and TGF‐β signaling. This is consistent with emerging literature demonstrating that RNA acetylation, primarily mediated by NAT10, stabilizes oncogenic transcripts [16, 17]. Conversely, low ac4C levels correlated with cell cycle and fatty acid metabolism, pointing to a more metabolically controlled, less invasive tumor state. One of our most notable discoveries lies in the TME architecture; high ac4C tumors exhibited dense stromal infiltration alongside a highly immunosuppressive signature (elevated LAG3, CD14, SIRPA) and macrophage/NK cell dominance. This finding implies that high ac4C modification orchestrates an “immune‐excluded” or “fibrotic” TME, which notoriously physically impedes immune cell infiltration and actively exhausts cytotoxic T cells [18].

Further elucidating the fibrotic nature of high‐ac4C tumors, scRNA‐seq analysis isolated CERCAM as a key microenvironmental effector. CERCAM (Cerebral Endothelial Cell Adhesion Molecule) has been sporadically reported in some cancers [19], but its exact cellular distribution in the CRC TME was previously ambiguous. Our single‐cell analysis definitively mapped CERCAM expression almost exclusively to CAFs, not tumor cells. The progressive hypermethylation of CERCAM DNA from normal to primary and then metastatic cells suggests a highly coordinated epigenetic adaptation during tumor spread. In our co‐culture models, manipulating CERCAM precisely in CAFs drastically altered the proliferative and invasive behaviors of the bystander CRC cells. This proves that ac4C networks do not just drive self‐renewal in epithelial cells, but prominently modulate stromal cells (like CAFs) to excrete paracrine signals or reorganize the ECM (supported by our GSVA findings of collagen fibril crosslinking), thereby pushing the tumor toward metastasis [20, 21]. Furthermore, by rigorously restricting our single‐cell analysis to high‐quality 10X Genomics datasets to eliminate platform‐specific biases, we revealed that CERCAM is not just a generic stromal marker. It is highly enriched in specific CAF subpopulations, particularly matrix‐remodeling CAFs (e.g., myCAFs). Since myCAFs are the primary drivers of collagen deposition and ECM crosslinking, this perfectly aligns with our GSVA findings that the high‐ac4C group is enriched in ECM receptor interactions.

The genomic and pharmacogenomic insights provided by the ac4C score carry substantial translational weight. The divergent mutation frequencies of APC and TTN between colon (COAD) and rectal (READ) cancers in the high ac4C group underscore the spatial heterogeneity of the colon and rectum, reminding clinicians that epigenomic drivers may interact differently with genomic foundations depending on the anatomical site [22]. Therapeutically, the prediction that high‐ac4C tumors are resistant to multi‐line chemotherapies aligns with their high‐stroma, EMT‐active phenotype, which typically limits drug penetration and promotes survival [23]. However, their increased sensitivity to targeted kinase inhibitors like pazopanib and gefitinib presents a lucrative alternative. Pazopanib targets angiogenesis and stromal signaling (VEGFR, PDGFR), making rational sense for a stroma‐heavy, CAF‐rich (high CERCAM) TME [24].

While this study is primarily rooted in molecular oncology and tumor microenvironment characterization, our findings hold meaningful translational implications for clinical nursing and holistic patient management. The development of an ac4C‐based prognostic risk score provides a potential novel stratification tool that could facilitate precision nursing. In clinical settings, identifying high‐risk patients based on their molecular profiles can empower oncology nurses to implement more intensive surveillance, early symptom management, and tailored psychological support.

Furthermore, our drug sensitivity predictions suggest that specific patient subgroups may exhibit distinct responses to targeted therapies (e.g., specific kinase inhibitors). Understanding these molecular‐based therapeutic vulnerabilities allows the nursing team to anticipate and proactively manage specific drug‐related toxicities and adverse events. By bridging the gap between molecular subtyping and clinical care, such predictive models can ultimately assist multidisciplinary teams, including advanced practice nurses, in designing more individualized, safe, and effective care pathways for colorectal cancer patients.

Our study has certain limitations. First, an apparent discrepancy was noted regarding CERCAM expression: while scRNA‐seq detected negligible CERCAM in epithelial cells, qPCR revealed its presence. This is likely due to the superior sensitivity of qPCR in detecting low‐abundance transcripts, combined with the stringent normalization processes inherent in scRNA‐seq that may mask minor epithelial expression. Moreover, long‐term cultured in vitro cell lines often diverge from in vivo transcriptomes. Second, our clinical validation cohort dichotomized patients into an uneven distribution (60 high vs. 10 low) based on optimal expression cutoffs. While statistically valid, this non‐random selection introduces potential sample bias, necessitating validation in larger, multi‐center, continuously distributed cohorts. Furthermore, any mechanistic link between ac4C RNA modification and the observed CERCAM DNA methylation remains strictly hypothetical and warrants future epigenetic cross‐talk studies. Additionally, our bioinformatic analyses suggest a strong correlation, but this does not constitute direct evidence of ac4C‐driven fibrosis. Additionally, drug sensitivity predictions based on algorithms like pRRophetic or oncoPredict can yield conflicting results depending on the database version, and therefore must be interpreted with caution as purely predictive inferences. Finally, the pharmacogenomic predictions generated via pRRophetic are derived from bulk transcriptomes. While useful for hypothesis generation, this algorithm has inherent limitations and cannot fully substitute for individualized in vitro or in vivo drug sensitivity assays, particularly for agents targeting the microenvironment [25].

## 5 Conclusion

In conclusion, this study establishes that ac4C RNA modification is not merely a tumor‐intrinsic epigenetic event, but a profound orchestrator of the CRC microenvironment. By computing an ac4C prognostic score, we demonstrated its utility in predicting aggressive clinical trajectories, fibrotic immune‐exclusion, and differential drug sensitivities. Crucially, single‐cell and in vitro validations unveiled CERCAM as a CAF‐specific effector within the ac4C network, driving pro‐tumorigenic stroma‐epithelium crosstalk. While the direct regulatory mechanisms warrant further molecular exploration, our findings highlight CAF‐derived CERCAM as a promising translational target to dismantle the immunosuppressive stroma in CRC.

## Author Contributions

**Xinyi Zhou:** conceptualization, supervision, project administration, resources. **Yang Xia:** methodology, writing – original draft, visualization, formal analysis. **Ruige Zhang:** resources, software, visualization, data curation. **Jialin Dai:** software, visualization, validation, investigation, writing – review and editing, data curation.

## Funding

The authors have nothing to report.

## Ethics Statement

This study was conducted in strict accordance with the ethical principles outlined in the Declaration of Helsinki. It was approved by the Ethics Committee of the Affiliated Hospital with Jiangnan University. As this study involves the analysis of human participant data and/or tissue samples, we confirm that informed consent was obtained from all individual participants included in the study. The informed consent process clearly informed the participants about the nature, purpose, potential risks, and benefits of the study, and the consent forms were personally signed by the participants or their legally authorized representatives.

## Consent

The subjects involved in this study have all signed written consent to participate in the research.

## Conflicts of Interest

The authors declare no conflicts of interest.

## Supporting information


**Figure S1:** Batch effect removal and visualization of ac4C‐based consensus clustering. (A and B) Principal component analysis (PCA) plots representing the sample distributions of the TCGA and combined GEO cohorts (A) before and (B) after the removal of batch effects using the ComBat algorithm. (C and D) Alternative visualization of cluster separation using PCA and t‐distributed stochastic neighbor embedding (t‐SNE) (D) in external validation datasets, confirming the distinct transcriptional profiles between ac4C Cluster 1 and Cluster 2.

**Figure S2:** Supplementary clinical and pathway characteristics of ac4C subgroups. (A and B) Additional validations showing the association of Cluster 2 with advanced N‐stage and M‐stage across external clinical datasets. (C) Comparison of ac4C modification ssGSEA scores between Cluster 1 and Cluster 2 in the combined GEO validation cohort. (D) Proportion of patients with high vs. low ac4C modification levels across advancing overall pathological stages based on the combined GEO database.

**Figure S3:** Comprehensive profiling of the tumor immune microenvironment across ac4C subgroups. (A) Detailed bar plots depicting the immune cell infiltration proportions estimated by CIBERSORT in external validation cohorts. (B) Heatmap showing the ssGSEA enrichment scores for diverse immune cell types and immune‐related functions across all included samples. (C) Validation of the significant differences in Stromal, Immune, and ESTIMATE scores between ac4C subgroups in an independent cohort. (D) Extended correlation network analysis illustrating the interplay between the ac4C score and a broader spectrum of TME cellular components. (E) Validation of immune checkpoint and marker gene expression differences (e.g., LAG3, PD‐L1, CD14, CD8A) between subgroups in external datasets.

**Figure S4:** Validation of the ac4C‐related prognostic risk signature in the independent combined GEO cohort. (A) Distribution of the risk scores and patient survival status in the combined GEO validation cohort (comprising GSE14333, GSE17538, GSE38832, and GSE39582). Patients were stratified into high‐ and low‐risk groups based on the median risk score. (B) Heatmap illustrating the expression patterns of the selected prognostic signature genes (including CERCAM) between the high‐risk and low‐risk groups in the validation cohort. (C) Kaplan–Meier survival analysis comparing the Overall Survival (OS) of colorectal cancer patients in the high‐ versus low‐risk groups. Statistical significance was determined using the two‐sided log‐rank test. (D) Time‐dependent Receiver Operating Characteristic (ROC) curves demonstrating the predictive accuracy of the risk signature. The Area Under the Curve (AUC) values for 1‐year, 3‐year, and 5‐year OS are annotated in the plot.

**Figure S5:** Estimation of chemotherapeutic and targeted drug sensitivity using the oncoPredict algorithm. (A) Boxplots comparing the estimated half‐maximal inhibitory concentration (IC_50_) values of representative therapeutic agents between the high‐risk and low‐risk groups. Drug sensitivities were predicted utilizing the updated oncoPredict R package based on the CTRP/GDSC reference datasets. Lower IC_50_ values indicate higher predicted drug sensitivity. Panels display the differential predicted responses for candidate agents, including Pazopanib, Gefitinib, and other top‐ranked compounds. The bold horizontal lines within the boxes represent the median values, and the whiskers extend to the most extreme data points within 1.5× interquartile range (IQR). Statistical differences between the two groups were evaluated using the Wilcoxon rank‐sum test (ns, not significant; *p < 0.05; **p < 0.01; ***p < 0.001).

**Table S1:** All CRC patients' clinical characteristics.

**Table S2:** ac4C related genes.

**Table S3:** DEGs between high ac4C cluster and high ac4C cluster.

**Table S4:** The gene model constituting ac4C score.

**Table S5:** Statistical tests of mutation landscape between ac4C_score_group.

## Data Availability

The data that support the findings of this study are available from the corresponding author upon reasonable request.
